# Integrating multi-source data and machine learning to Decipher the psoriasis-COPD comorbidity

**DOI:** 10.1007/s10238-026-02065-y

**Published:** 2026-02-07

**Authors:** YuFeng He, LinMei Xiang, YanCheng He, GuanJie Wang, HuiLi Jiang, YuZe Li, XiaoYi Qi

**Affiliations:** 1 Department of Dermatology, The Affiliated Hospital, Southwest Medical University, Luzhou, Sichuan China; 2 Skin Structure and Function Key Laboratory of Luzhou, Department of Dermatology, The Affiliated Hospital, Southwest Medical University, Luzhou, China

**Keywords:** Psoriasis, COPD, Inflammatory Pathways, Biomarkers, Machine Learning, Comorbidity, Risk Factors, Diagnostic Model

## Abstract

The epidemiological and molecular associations between psoriasis and chronic obstructive pulmonary disease (COPD) remain incompletely elucidated. To explore this association and shared mechanisms, this study integrated data of the National Health and Nutrition Examination Survey (NHANES) 2003–2014 (*n* = 17,416), assessing this association via multivariable logistic regression and subgroup analysis. Transcriptomic data of psoriasis (skin tissue) and COPD (alveolar macrophages) were retrieved from the Gene Expression Omnibus (GEO) database. Candidate biomarkers were identified via differentially expressed gene (DEG) analysis, weighted gene coexpression network analysis (WGCNA), and machine learning [Random Forest (RF) and least absolute shrinkage and selection operator (LASSO)], followed by validation of their diagnostic efficacy. In the fully weighted and adjusted model, no statistically significant association was found between psoriasis and COPD (OR = 1.25, 95% CI: 0.93–1.68, *p* = 0.14), although trend-level associations were observed among smokers, individuals with hypertension, and those with unstable marital status. We identified 85 shared differentially expressed genes (DEGs), enriched in inflammatory pathways such as the chemokine signaling pathway, and screened three candidate genes (UCK2, P4HA1, and HIBADH). A RF diagnostic model based on these genes achieved Area Under the Curves (AUCs) of 0.935 for psoriasis and 0.962 for COPD in external validation sets. These findings suggest that the comorbidity between psoriasis and COPD may be influenced by risk factors such as smoking and hypertension, as well as shared inflammatory pathways and differentially expressed genes (DEGs) regulation. Psoriasis could serve as a potential window for early COPD screening and provide novel cross-disease therapeutic targets.

## Introduction

Psoriasis is a chronic, relapsing, inflammatory skin disease driven by genetic susceptibility and environmental factors. With a global prevalence exceeding 125 million, it represents a pressing global public health concern [1, 2]. It is characterized by scaly erythematous plaques that can be localized or widespread, significantly affecting patients’ appearance. Importantly, psoriasis is not merely a cutaneous disorder. It is frequently associated with metabolic and cardiovascular comorbidities such as stroke, hypertension, and diabetes, and can coexist with conditions like inflammatory bowel disease, arthritis, and anxiety-depressive disorders. These associations inflict a dual burden on both physical health and psychological well-being, severely diminishing quality of life [3–5]. Furthermore, the chronic relapsing nature of the disease and the need for comorbidity management substantially increase healthcare utilization and societal costs [6–8].

Chronic obstructive pulmonary disease (COPD) ranks among the most common chronic respiratory diseases worldwide and is the fourth leading cause of death globally. It is defined by persistent respiratory symptoms and progressive airflow limitation, with chronic airway inflammation and aberrant immune activation constituting its core pathophysiological processes [9–11]. This sustained inflammatory state triggers the infiltration of various immune cells and the release of inflammatory mediators, driving airway remodeling and parenchymal destruction while further propelling disease progression through immune dysregulation [12–14]. Although early intervention is effective, significant gaps remain in the early diagnosis and management of COPD. Many patients are first diagnosed at severe or very severe stages, which exacerbates disease burden, complicates treatment, and increases the associated socioeconomic costs [15, 16].

As two chronic inflammatory conditions with high disease burdens, psoriasis and COPD share core pathological features of immune system dysregulation and persistent chronic inflammation [4, 5, 11]. In clinical practice, thorough skin examinations of psoriasis patients often reveal that a considerable proportion present with barrel chest, a characteristic thoracic deformity associated with COPD. Furthermore, several studies have suggested a potential association between COPD and psoriasis [17, 18]. However, systematic analyses based on large-scale population data remain scarce, and the exploration of their potential shared molecular mechanisms is still in its infancy.

Therefore, this study aimed to systematically investigate the association between psoriasis and COPD and its underlying mechanisms by integrating multi-source data. Specifically, we first utilized nationally representative data from the National Health and Nutrition Examination Survey (NHANES) to evaluate the epidemiological association between the two diseases after adjusting for a range of potential confounders. Subsequently, we performed bioinformatic mining of transcriptomic data for psoriasis (skin tissue) and COPD (alveolar macrophages) from the public Gene Expression Omnibus (GEO) database. This involved identifying shared differentially expressed genes and pathways, and applying machine learning methods to screen candidate genes. Our goal was to provide preliminary evidence and research clues for understanding their comorbidity from both macroscopic (epidemiological) and microscopic (molecular) perspectives.

## Methods

### Cross-Sectional epidemiological analysis based on the NHANES database

#### Data source and study population

This study leveraged publicly available data of the National Health and Nutrition Examination Survey (NHANES). We integrated data from five two-year cycles (spanning ten years): 2003–2004, 2005–2006, 2009–2010, 2011–2012, and 2013–2014. NHANES employs a complex, multistage, probability sampling design to obtain a nationally representative sample of the non-institutionalized civilian population in the United States. All participants provided written informed consent, and the survey protocol was approved by the National Center for Health Statistics (NCHS) Ethics Review Board.

#### Variable definitions

Definition of COPD: COPD was defined based on meeting either of the following criteria: (1) a post-bronchodilator ratio of forced expiratory volume in the first second to forced vital capacity (FEV₁/FVC) of less than 0.70; or  (2) a self-reported, physician-diagnosed history of emphysema, chronic bronchitis, or chronic obstructive pulmonary disease (COPD) from a standardized questionnaire [19, 20]. Definition of Psoriasis: Psoriasis was defined based on responses to the “Medical Conditions” questionnaire. Specifically, participants were considered to have psoriasis if they answered “Yes” to the question “Has a doctor ever informed you that you have psoriasis?” and confirmed the diagnosis was given by a doctor or other health professional [21–23]. Covariates: Selected covariates included demographic characteristics (age, sex [male, female], marital status, education level [less than high school, high school graduate, college or above, other], race/ethnicity [Non-Hispanic White, Non-Hispanic Black, Mexican American, Other], and family income-to-poverty ratio [PIR]), disease-related factors (hypertension, diabetes, cancer), and lifestyle/health-related factors (body mass index [BMI], smoking status [defined as having smoked at least 100 cigarettes in one’s lifetime], and alcohol use [defined as having consumed at least 12 alcoholic drinks in the past year]). Diagnoses of diabetes, hypertension, and cancer were based on self-report of a physician’s diagnosis.

#### Statistical analysis

All analyses followed the NHANES analytical guidelines. Survey weights were applied to generate estimates representative of the non-institutionalized U.S. population, accounting for the complex sampling design including clustering and stratification. When combining multiple survey cycles, the recommended weight adjustment procedures from NCHS were applied. The participant inclusion and exclusion flowchart was first described. For the final analytical sample, characteristics were stratified by COPD status. Continuous variables are presented as mean ± standard deviation (SD), and categorical variables as number (percentage). The association between psoriasis and COPD was assessed using multivariable logistic regression models under both unweighted and weighted conditions. Three levels of adjustment were employed: unadjusted, demographically adjusted (for age, sex, race/ethnicity, education, marital status, PIR), and fully adjusted (further including BMI, smoking, alcohol use, hypertension, diabetes, and cancer history). Subgroup analyses based on weighted data were performed for key covariates. All analyses were conducted using R software (version 4.5.0), with a two-sided P-value < 0.05 considered statistically significant.

### Bioinformatics analysis based on the GEO database

#### Data acquisition and preprocessing

Four microarray datasets were downloaded from the Gene Expression Omnibus (GEO; http://www.ncbi.nlm.nih.gov/geo). The selection criteria were: containing gene expression profiles from patients with either psoriasis or COPD and healthy controls, with a relatively large sample size and consistent platform. Training Sets: Psoriasis: GSE13355 (platform GPL570), containing 58 psoriasis patients (skin tissue) and 64 healthy controls. COPD: GSE130928 (platform GPL570), containing 22 COPD patients (alveolar macrophages) and 24 healthy controls. External Validation Sets: Psoriasis: GSE14905 (platform GPL570), containing 33 patients and 21 controls (skin tissue). COPD: GSE13896 (platform GPL570), containing 12 patients and 24 controls (alveolar macrophages). All raw data were preprocessed using the R package limma for background correction, quantile normalization, and log₂ transformation.

#### Identification of differentially expressed genes (DEGs)

The limma package was used to identify DEGs between disease and control groups within each training set. Genes with a false discovery rate (FDR) adjusted P-value < 0.05 and an absolute log₂ fold change (|log₂FC|) > 0.5 were considered significant DEGs. Venn diagrams were used to identify shared DEGs that were either co-upregulated or co-downregulated in both psoriasis and COPD.

#### Functional and pathway enrichment analysis

The clusterProfiler R package was utilized to perform Gene Ontology (GO) enrichment analysis (including Biological Process [BP], Cellular Component [CC], and Molecular Function [MF] categories) and Kyoto Encyclopedia of Genes and Genomes (KEGG) pathway enrichment analysis on the shared DEGs. An FDR < 0.05 was set as the significance threshold. The results were visualized using the ggplot2 package.

#### Weighted gene co-expression network analysis (WGCNA)

The WGCNA R package was used to construct gene co-expression networks for the psoriasis and COPD training sets separately. The procedure included: (1) Sample clustering and outlier detection/removal;  (2) Selection of an appropriate soft-thresholding power (β) to achieve a scale-free topology (scale-free topology fit index R² > 0.8);  (3)  Module identification using the dynamic tree cut algorithm with a minimum module size of 30, followed by merging of modules with a correlation > 0.25;  (4) Calculation of the correlation between module eigengenes (MEs) and the disease phenotype (case/control). Modules significantly associated with the disease (|correlation coefficient| > 0.5 and *P* < 0.05) were selected;  (5)  Extraction of genes shared between the disease-associated modules from both datasets.

#### Candidate gene screening and validation

To screen for robust candidate biomarkers, two machine learning algorithms were combined: Random Forest (RF): The randomForest package was used to build classification models. The importance score (Mean Decrease Gini) for each gene was calculated. Genes with an importance score > 1 in either the psoriasis or COPD dataset were retained. Least Absolute Shrinkage and Selection Operator (LASSO) Regression: The glmnet package was used for feature selection. The optimal regularization parameter (λ) was determined via 10-fold cross-validation, and genes with non-zero coefficients at this λ were retained. The intersection of genes identified by both RF and LASSO in each disease dataset was defined as preliminary candidate genes. Expression Validation and Diagnostic Performance Evaluation: Independent sample t-tests were used to compare the expression levels of candidate genes between disease and control groups in both training and external validation sets. The pROC package was used to plot receiver operating characteristic (ROC) curves and calculate the area under the curve (AUC) to evaluate the ability of individual genes to discriminate disease from control. An AUC > 0.7 was considered indicative of preliminary diagnostic value. Model Selection: Eight machine learning models were constructed based on the candidate genes: Random Forest (RF), Decision Tree (DTS), Logistic Regression, K-Nearest Neighbors (KNN), XGBoost, Gradient Boosting Machine (GBM), Neural Network, and glmBoost. Finally, a comprehensive Random Forest diagnostic model was built using the expression values of all candidate genes, and its combined AUC was calculated in the validation sets. SHapley Additive exPlanations (SHAP) values were used to interpret feature importance and visualize the contribution of each candidate gene to the model’s predictions.

## Results

### Results of the NHANES cross-sectional survey

#### Study design and sample characteristics

**Fig. 1:**
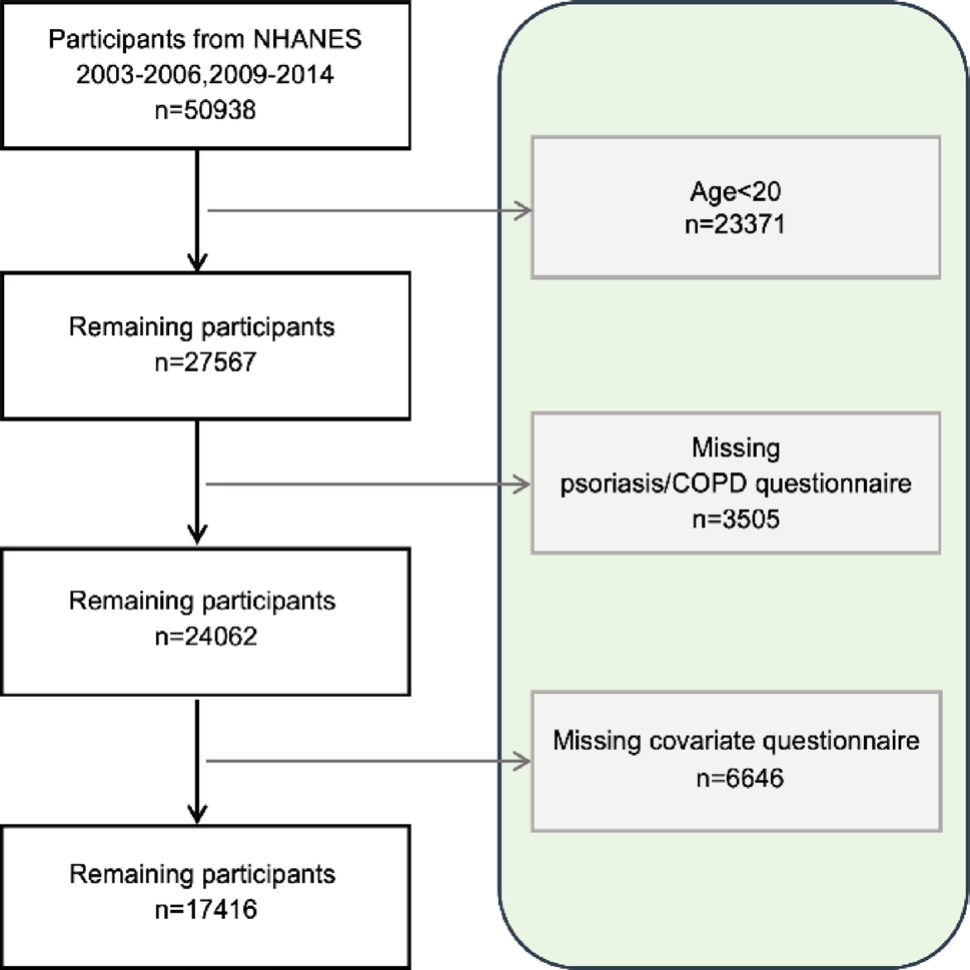
Flowchart of participant selection in this study. Data source: National Health and Nutrition Examination Survey (NHANES)

This analysis was based on data from the National Health and Nutrition Examination Survey (NHANES) cycles 2003–2006 and 2009–2014. The participant selection process was as follows: Initially, 50,938 participants were included. After excluding 23,371 individuals aged < 20 years, 27,567 remained. Further exclusion of participants with missing psoriasis/COPD questionnaire data (*n* = 3,505) or missing covariate information (*n* = 6,646) yielded a final analytical sample of 17,416 participants (Fig. 1).

#### Baseline characteristics

Weighted baseline characteristics stratified by COPD status are presented in Table 1. COPD patients differed significantly from non-COPD participants in most characteristics. COPD patients were older (mean age 51.56 ± 15.63 years vs. 42.60 ± 14.56 years, *p* < 0.001) and had a higher proportion of females (53.8% vs. 50.1%, *p* = 0.045). They also had a higher prevalence of unstable marital status (38.3% vs. 35.2%, *p* = 0.029), lower educational attainment (*p* < 0.001), and a greater proportion with low income (PIR < 1) (17.4% vs. 13.6%, *p* < 0.001). Furthermore, the COPD group had a higher average BMI (29.41 ± 7.29 vs. 28.68 ± 6.73, *p* < 0.001), a higher prevalence of psoriasis (4.4% vs. 2.8%, *p* < 0.001), and a significantly higher proportion of smokers (66.0% vs. 42.6%, *p* < 0.001). The prevalences of comorbid diabetes (13.6% vs. 6.8%), hypertension (41.7% vs. 25.6%), and cancer (14.9% vs. 6.9%) were all higher in the COPD group (all *p* < 0.001). Regarding race/ethnicity, COPD patients had a higher proportion of Non-Hispanic White individuals (78.4% vs. 68.2%, *p* < 0.001) and a lower proportion of Mexican Americans (6.2% vs. 13.9%, *p* < 0.001).

**Table 1:** The baseline characteristics of participants enrolled in the NHANES cycles spanning from 2003–2006 and 2009–2014

Variables	No (*n*=15167)	Yes (*n*=2249)	*P*-value
**Age**			<0.001
Mean ± SD	42.60 ± 14.56	51.56 ± 15.63	
**BMI**			<0.001
Mean ± SD	28.68 ± 6.73	29.41 ± 7.29	
**Psoriasis**			<0.001
No	14,802 (97.2%)	2140 (95.6%)	
Yes	365 (2.8%)	109 (4.4%)	
**Gender**			0.045
Male	7430 (49.9%)	1081 (46.2%)	
Female	7737 (50.1%)	1168 (53.8%)	
**Education**			<0.001
Lower than high school education	3211 (14.0%)	576 (18.6%)	
Higher than high school education	8571 (64.0%)	1122 (57.4%)	
High school graduate	3385 (22.0%)	551 (24.0%)	
**Marital status**			0.029
Unstable marriage group	5864 (35.2%)	993 (38.3%)	
Stable marital status group	9303 (64.8%)	1256 (61.7%)	
**Race**			<0.001
Non-Hispanic white	6656 (68.2%)	1327 (78.4%)	
Non-Hispanic black	3332 (11.5%)	468 (9.4%)	
Mexican American	3703 (13.9%)	302 (6.2%)	
Other	1476 (6.4%)	152 (6.0%)	
**PIR**			<0.001
Poverty (PIR<1)	3108 (13.6%)	590 (17.4%)	
Low to middle income (1≤PIR≤3)	5886 (33.6%)	941 (38.4%)	
High income (PIR>3)	6173 (52.8%)	718 (44.2%)	
**Smoking**			<0.001
No	8858 (57.4%)	747 (34.0%)	
Yes	6309 (42.6%)	1502 (66.0%)	
**Diabetes**			<0.001
No	13,804 (93.2%)	1874 (86.4%)	
Yes	1363 (6.8%)	375 (13.6%)	
**Cancer**			<0.001
No	15,234 (93.1%)	1942 (85.1%)	
Yes	917 (6.9%)	307 (14.9%)	
**Hypertension**			<0.001
No	10,997 (74.4%)	1222 (58.3%)	
Yes	4170 (25.6%)	1027 (41.7%)	
**Alcohol**			<0.001
No	4089 (22.8%)	489 (18.3%)	
Yes	11,078 (78.2%)	1760 (81.7%)	

#### Association between psoriasis and COPD

The association was assessed using multivariable logistic regression models under unweighted and weighted conditions, with three levels of adjustment (unadjusted, demographically adjusted, fully adjusted). Unweighted Models (Table 2): Without adjustment, psoriasis was significantly associated with COPD (OR = 2.07, 95% CI: 1.65–2.56, *p* < 0.001). After adjusting for demographic factors, the association attenuated but remained significant (OR = 1.55, 95% CI: 1.22–1.96, *p* < 0.001). In the fully adjusted model, the association was still significant (OR = 1.22, 95% CI: 1.22–1.96, *p* = 0.0002). Weighted Models (Table 3): In the unadjusted weighted model, psoriasis was significantly associated with COPD (OR = 1.58, 95% CI: 1.20–2.09, *p* = 0.002). The association remained significant after demographic adjustment (OR = 1.38, 95% CI: 1.03–1.87, *p* = 0.03). However, in the fully adjusted weighted model (further adjusting for BMI, smoking, alcohol use, hypertension, diabetes, and cancer history), the association was no longer statistically significant (OR = 1.25, 95% CI: 0.93–1.68, *p* = 0.14).

**Table 2:** Unweighted associations between COPD and psoriasis in NHANES 2003–2006, 2009–2014 using multivariable logistic regression models

Variables	Crude model	Partly adjusted	Fully adjusted
COPD			
Non-Psoriasis	Ref.	Ref.	Ref.
Psoriasis	2.07 (1.65, 2.56) < 0.001	1.73 (1.37, 2.17) < 0.001	1.55 (1.22, 1.96) < 0.001

COPD: chronic obstructive pulmonary disease

Crude model: no covariate was adjusted

Partly adjusted: adjusted for gender, age, race, education, marital status, poverty-to-income ratio

Fully adjusted: adjusted for gender, age, race, education, marital status, poverty-to-income ratio, body mass index, smoking, alcohol, history of hypertension, diabetes and cancer

**Table 3:** Weighted associations between COPD and psoriasis in NHANES 2003–2006, 2009–2014 using multivariable logistic regression models

Variables	Crude model	Partly adjusted	Fully adjusted
COPD			
Non-Psoriasis	Ref.	Ref.	Ref.
Psoriasis	1.58 (1.20,2.09) 0.002	1.38 (1.03, 1.87)0.03	1.25 (0.93, 1.68)0.14

COPD: chronic obstructive pulmonary disease

Crude model: no covariate was adjusted

Partly adjusted: adjusted for gender, age, race, education, marital status, poverty-to-income ratio

Fully adjusted: adjusted for gender, age, race, education, marital status, poverty-to-income ratio, body mass index, smoking, alcohol, history of hypertension, diabetes and cancer

#### Subgroup analysis

**Fig. 2:**
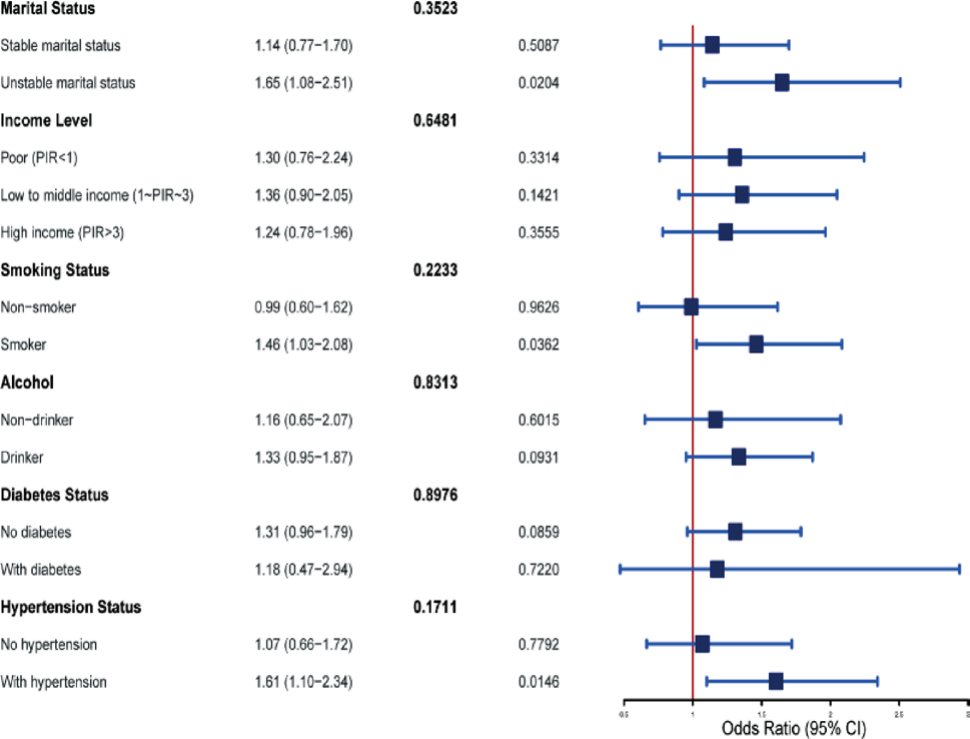
Forest plot of subgroup analyses for the association between psoriasis and COPD

Results of the subgroup analysis are shown in Fig. 2. The association between psoriasis and COPD remained statistically significant in subgroups with unstable marital status (OR = 1.65, 95% CI: 1.08–2.51, *p* = 0.0204), smokers (OR = 1.46, 95% CI: 1.03–2.08, *p* = 0.0362), and individuals with hypertension (OR = 1.61, 95% CI: 1.10–2.34, *p* = 0.0146). In contrast, the association was not significant in subgroups with stable marital status, non-smokers, or those without hypertension (all *p* > 0.05). The P-values for interaction tests for all subgroup variables were > 0.05, suggesting that the differences across subgroups did not reach statistical significance for interaction.

### Results of the GEO database analysis at the gene level

#### Differential expression gene analysis

**Fig. 3:**
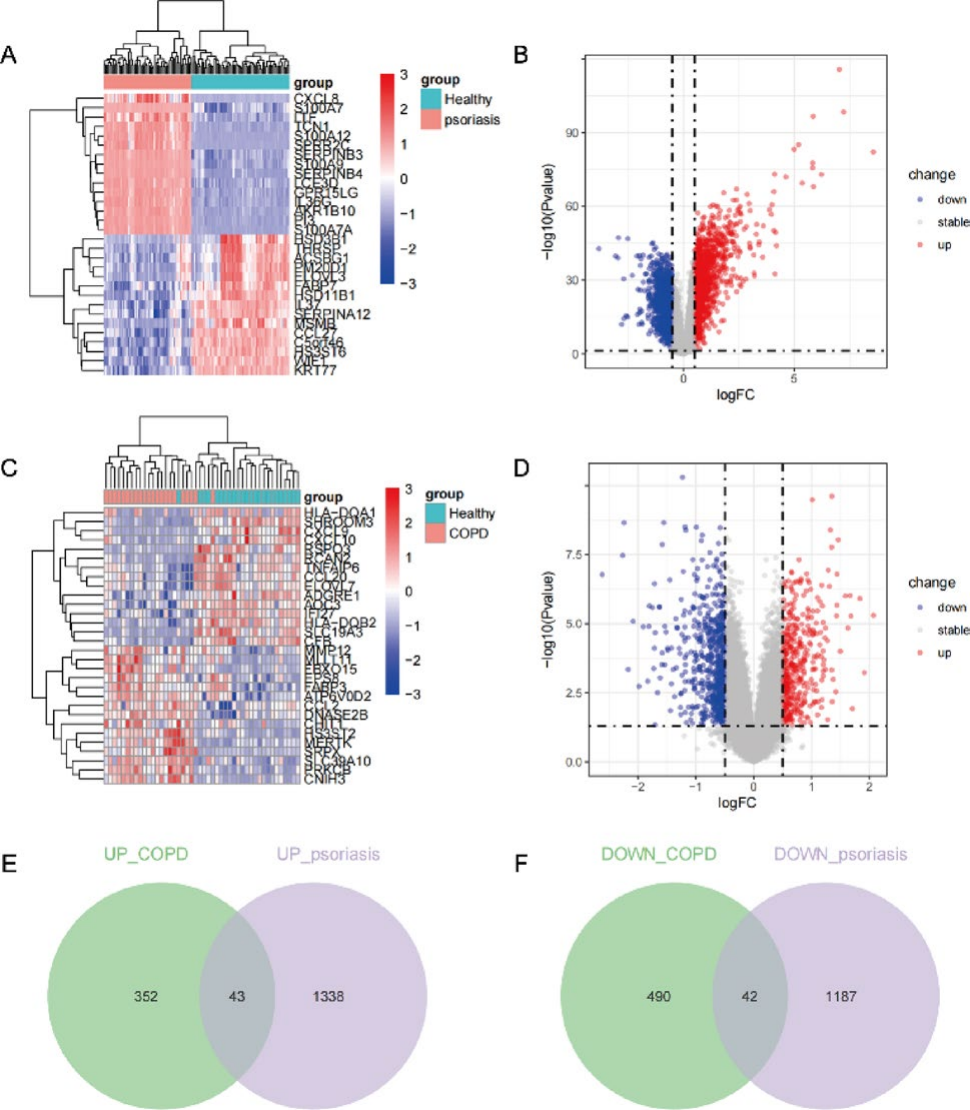
Identification of differentially expressed genes (DEGs) in patients with psoriasis (GSE13355) and chronic obstructive pulmonary disease (COPD, GSE130928). (**A**,** C**) Heatmaps of the top 30 DEGs in GSE13355 and GSE130928. (**B**,** D**) Volcano plots of all DEGs in GSE13355 and GSE130928. Blue dots represent downregulated DEGs, and red dots represent upregulated DEGs. (**E**,** F**) Venn diagrams illustrating co-upregulated and co-downregulated DEGs

In the training sets, the psoriasis dataset (GSE13355) yielded 2,600 DEGs (1,381 upregulated and 1,229 downregulated), and the COPD dataset (GSE130928) yielded 927 DEGs (395 upregulated and 532 downregulated), using the criteria of |log₂FC| > 0.5 and adjusted *P* < 0.05. The expression patterns of these DEGs are visualized in heatmaps and volcano plots (Fig. 3A-D). A total of 85 overlapping DEGs were identified between psoriasis and COPD, including 43 co-upregulated and 42 co-downregulated genes (Fig. 3E-F).

#### Functional enrichment analysis

**Fig. 4:**
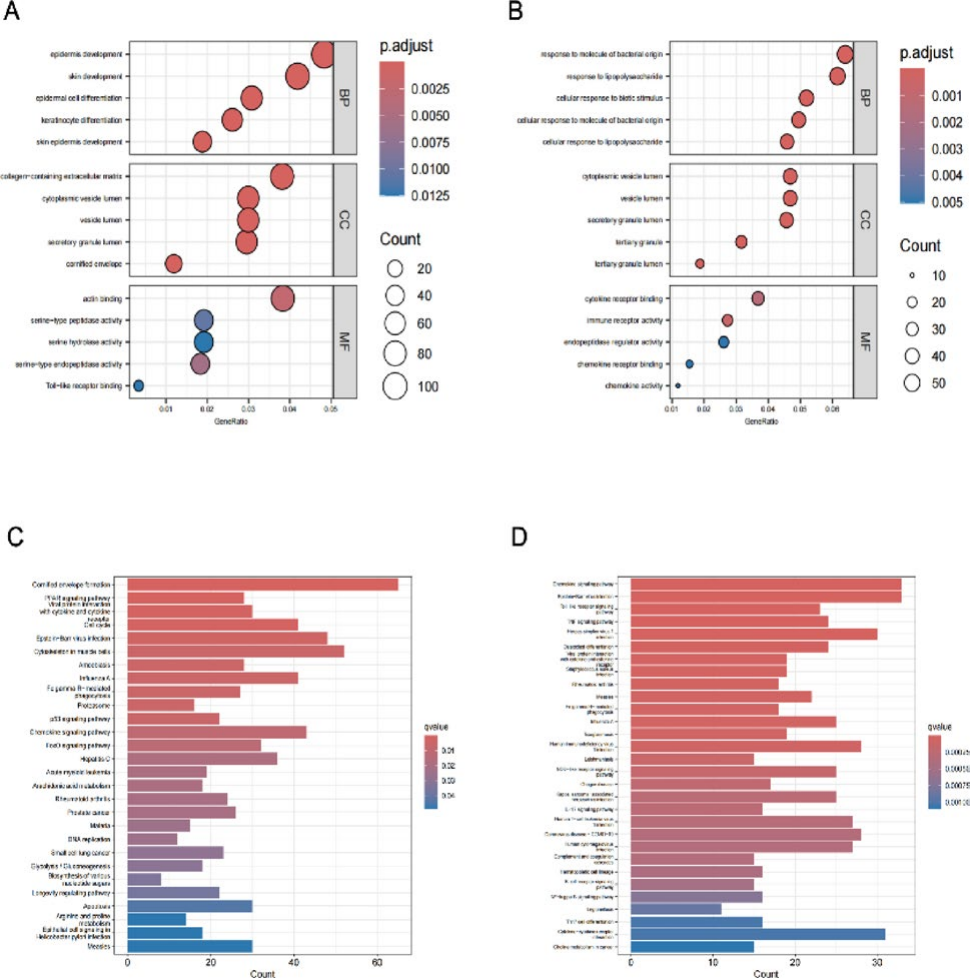
Enrichment analyses. (**A**,** B**) Bubble plots of Gene Ontology (GO) enrichment analyses. (**C**,** D**) Bar charts of Kyoto Encyclopedia of Genes and Genomes (KEGG) pathway enrichment analyses

GO enrichment analysis showed that the shared DEGs were significantly enriched in terms such as “cytoplasmic vesicle lumen” (CC), “response to biotic stimulus” (BP), and “chemokine receptor binding” (MF) (Fig. 4A-B). KEGG pathway analysis indicated that the shared DEGs were primarily enriched in pathways like the chemokine signaling pathway, Fc gamma R-mediated phagocytosis, and rheumatoid arthritis (Fig. 4C-D).

#### Weighted gene co-expression network analysis results

**Fig. 5:**
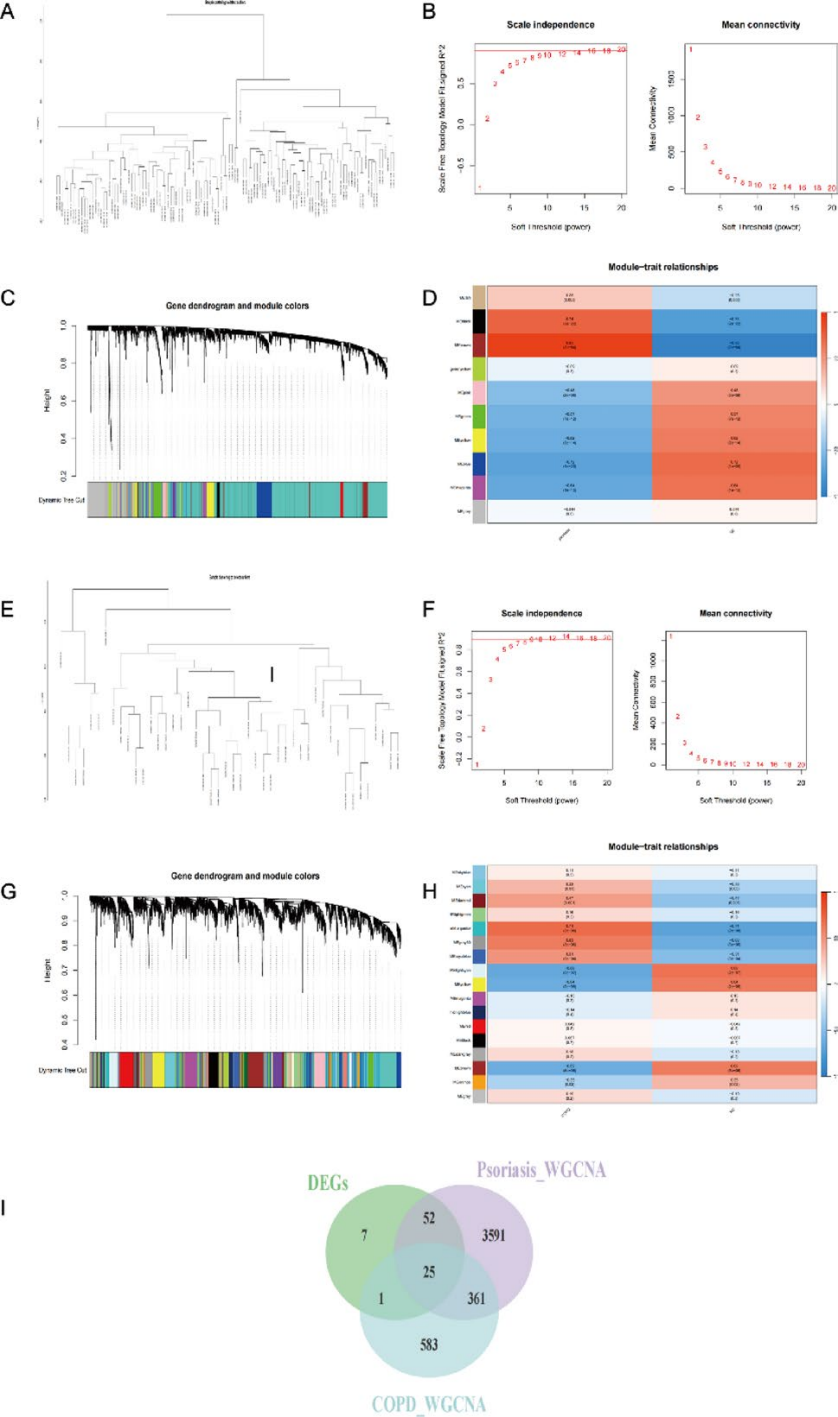
Weighted gene co-expression network analysis (WGCNA) of significant modules in psoriasis (GSE13355) and COPD (GSE130928). (**A**,** E**) Sample clustering dendrograms for GSE13355 (psoriasis) and GSE130928 (COPD). (**B**,** F**) Selection of soft-thresholding power in GSE13355 and GSE130928. (**C**,** G**) Gene clustering dendrograms generated by dynamic tree-cutting algorithm for GSE13355 and GSE130928. (**D**,** H**) Heatmaps of correlations between modules and clinical traits in GSE13355 and GSE130928. (**I**) Venn diagram showing the intersection of shared genes identified by WGCNA and DEGs

Key steps of the WGCNA are shown in Fig. 5. Sample clustering and outlier detection were performed first (Fig. 5A, E). By selecting the optimal soft-thresholding power (β = 12 for psoriasis, β = 7 for COPD), co-expression networks satisfying a scale-free topology were constructed (Fig. 5B, F). Gene modules were identified using the dynamic tree cut algorithm (minimum module size = 30) and a merge threshold of 0.25 (Fig. 5C, G). Disease phenotype-significantly associated modules were identified in both datasets (Fig. 5D, H). In psoriasis, the black (*r* = 0.74) and brown (*r* = 0.95) modules showed strong positive correlations with the disease, while the yellow, magenta, and blue modules showed negative correlations (|r|>0.6). In COPD, the darkturquoise (*r* = 0.71) and grey60 (*r* = 0.63) modules were positively correlated with the disease, whereas the lightcyan, yellow, and brown modules were negatively correlated (|r|>0.6). From these disease-significant modules, we extracted 25 shared DEGs (Fig. 5I), suggesting their potential involvement in the common regulatory processes of both diseases.

#### Candidate gene screening results based on machine learning

**Fig. 6:**
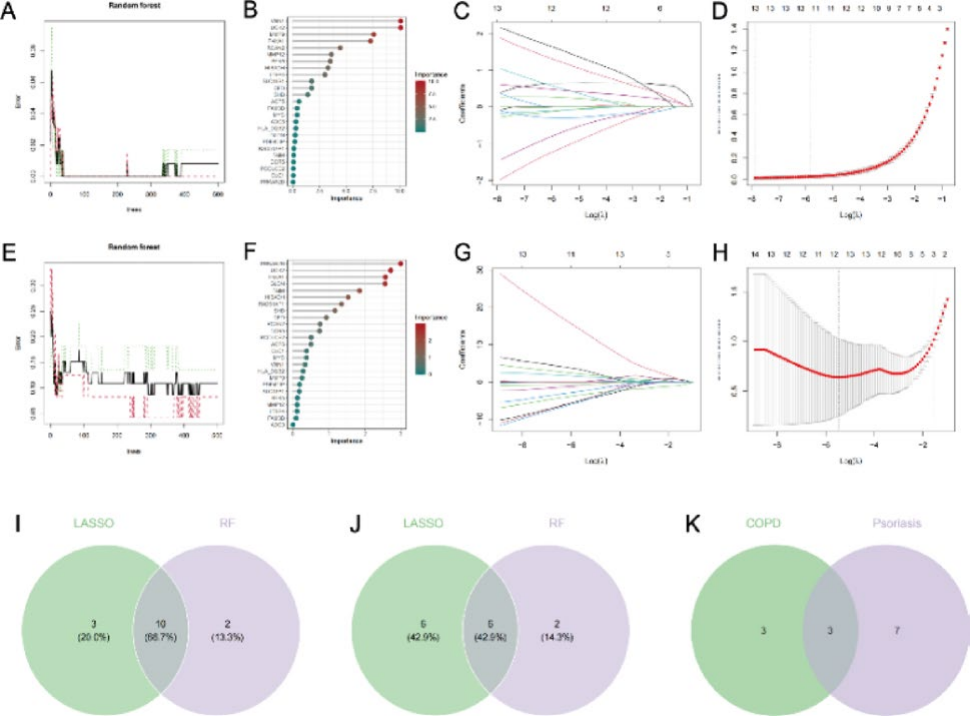
Identification of candidate biomarkers for psoriasis (GSE13355) and chronic obstructive pulmonary disease (GSE130928) via machine learning. (**A**,** B**) Random forest learning curve and bar chart of relative gene importance scores for psoriasis (GSE13355) (**C**,** D**) LASSO regression coefficient trajectory plot and deviance-regularization parameter plot for psoriasis (GSE13355) (**E**,** F**) Random forest learning curve and bar chart of relative gene importance scores for chronic obstructive pulmonary disease (GSE130928) (**G**,** H**) LASSO regression coefficient trajectory plot and deviance-regularization parameter plot for chronic obstructive pulmonary disease (GSE130928), (**I**) Venn diagram showing 10 candidate diagnostic genes identified for psoriasis (GSE13355) by LASSO regression and random forest (RF) algorithms, (**J**) Venn diagram showing 6 candidate diagnostic genes identified for chronic obstructive pulmonary disease (GSE130928) by LASSO regression and random forest (RF) algorithms, (**K**) Venn diagram showing 3 overlapping candidate diagnostic genes between psoriasis (GSE13355) and chronic obstructive pulmonary disease (GSE130928)

We integrated two machine learning algorithms for feature gene screening. The analytical results are shown in Fig. 6: the error trend and gene importance scores from the Random Forest (RF) model (Fig. 6A-B), and the coefficient trajectory and cross-validation curve from the LASSO regression for the psoriasis dataset (Fig. 6C-D); corresponding results for the COPD dataset are shown in Fig. 6E-H. In the RF analysis, genes with an importance score > 1 were retained. In the LASSO analysis, genes with non-zero coefficients at the optimal λ value determined by cross-validation were retained. Taking the intersection of results from both methods within each dataset yielded 10 overlapping genes for psoriasis (Fig. 6I) and 6 for COPD (Fig. 6J). Finally, the intersection of these two gene sets identified three candidate genes: UCK2, P4HA1, and HIBADH (Fig. 6K).

#### Diagnostic performance validation of candidate genes

**Fig. 7:**
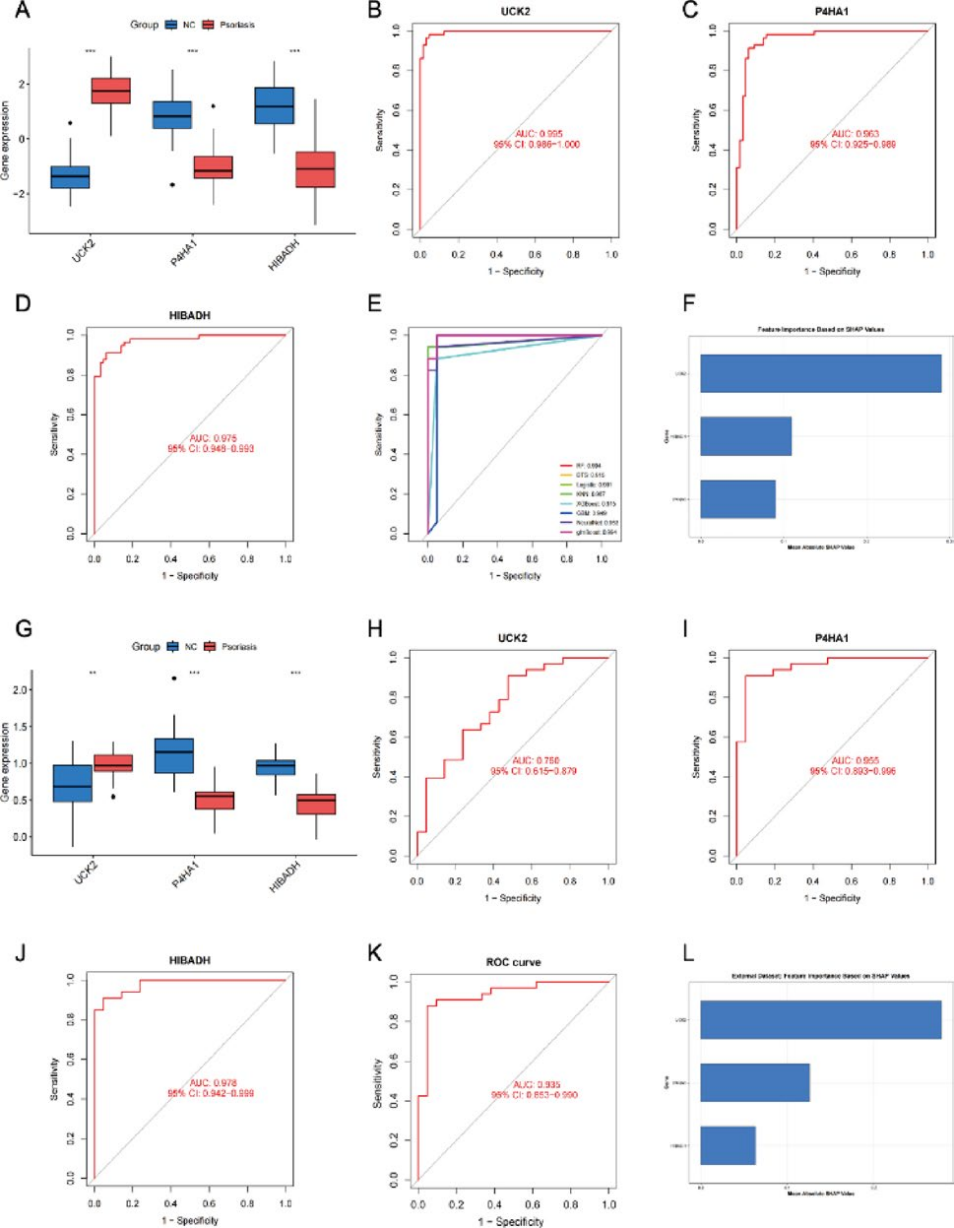
Psoriasis dataset (GSE13355) and external validation set (GSE14905). (**A**,** G**) Expression levels of UCK2, P4HA1, and HIBADH in GSE13355 and GSE14905. *P < 0.05; **P < 0.01; ***P < 0.001; ****P < 0.0001; ns: not significant. (**B-E**) ROC curves of UCK2, P4HA1, HIBADH, and 8 machine learning models in psoriasis (GSE13355). (**K**) ROC curves of UCK2, P4HA1, HIBADH, and the random forest model in the external validation set (GSE14905). (**F**,** L**) Bar charts of feature importance based on SHAP values in the random forest model for GSE13355 and GSE14905

**Fig. 8:**
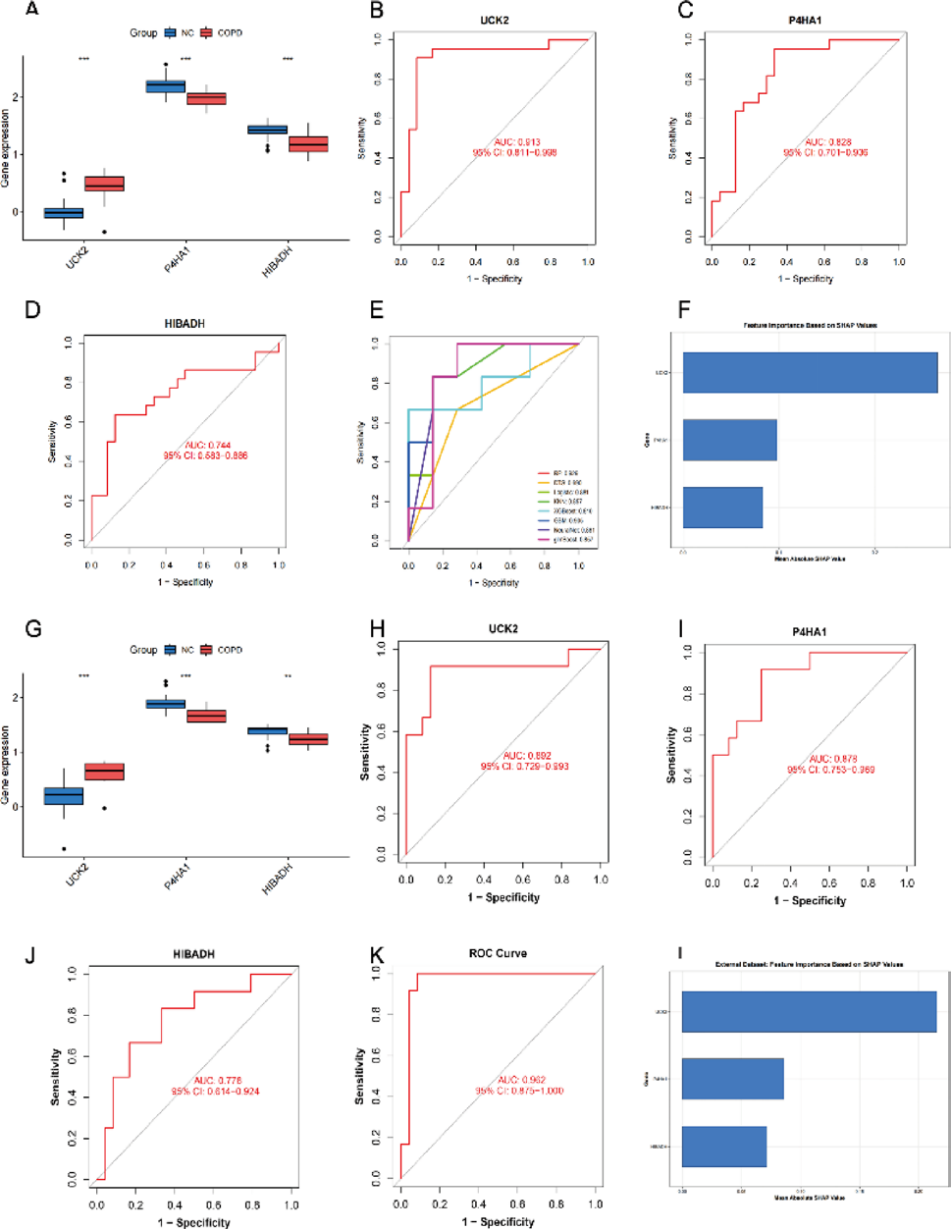
COPD dataset (GSE130928) and external validation set (GSE13896). (**A**,** G**) Expression levels of UCK2, P4HA1, and HIBADH in GSE130928 and GSE13896. *P < 0.05; **P < 0.01; ***P < 0.001; ****P < 0.0001; ns: not significant. (**B-E**) ROC curves of UCK2, P4HA1, HIBADH, and 8 machine learning models in COPD (GSE130928). (**H-K**) ROC curves of UCK2, P4HA1, HIBADH, and the random forest model in the external validation set (GSE13896). (**F**,** L**) Bar charts of feature importance based on SHAP values in the random forest model for GSE130928 and GSE13896

In both training sets (Figs. 7A and 8A) and external validation sets (Figs. 7E and 8E), UCK2 expression was consistently and significantly upregulated in disease groups, whereas P4HA1 and HIBADH were consistently downregulated (all *P* < 0.01). In the training sets, the AUCs of the ROC curves for the three genes in distinguishing disease from healthy controls all exceeded 0.7 (Figs. 7B-D and 8B-D). Specifically, for psoriasis, the AUCs were 0.995 for UCK2, 0.963 for P4HA1, and 0.975 for HIBADH. For COPD, the AUCs were 0.913 for UCK2, 0.828 for P4HA1, and 0.744 for HIBADH. Eight machine learning models were constructed based on these three genes for disease classification: Random Forest (RF), Decision Tree (DTS), Logistic Regression, K-Nearest Neighbors (KNN), XGBoost, Gradient Boosting Machine (GBM), Neural Network, and glmBoost (Figs. 7E and 8E). Based on the highest AUC achieved on the training set, the RF model was selected as the optimal model. SHapley Additive exPlanations (SHAP) value analysis revealed the contribution of each feature in the RF models. In the RF diagnostic models constructed for psoriasis and COPD separately, UCK2 had the highest mean absolute SHAP value (Figs. 7F and 8F). In the independent external validation sets, the three genes maintained diagnostic discrimination ability with AUCs > 0.7 (Figs. 7H-J and 8H-J). Specifically, in the psoriasis validation set (GSE14905), the AUCs were 0.760 for UCK2, 0.955 for P4HA1, and 0.978 for HIBADH. In the COPD validation set (GSE13896), the AUCs were 0.892 for UCK2, 0.878 for P4HA1, and 0.778 for HIBADH. Furthermore, the RF diagnostic model built on the three genes achieved AUCs of 0.935 (for psoriasis) and 0.962 (for COPD) in the respective external validation sets (Figs. 7K and 8K). UCK2 consistently demonstrated the highest mean absolute SHAP value in these final models (Figs. 7L and 8L).

## Discussion

This study integrated large-scale population data from NHANES and transcriptomic data from GEO to explore the comorbidity association between psoriasis and COPD and its underlying mechanisms from both macro-epidemiological and micro-molecular perspectives. Our findings indicate that the association between psoriasis and chronic obstructive pulmonary disease (COPD) is modulated by confounding factors such as smoking, obesity, hypertension, and marital instability. After adjusting for these factors, the overall association strength was attenuated, a result consistent with some previous studies, suggesting that these factors may be important confounders influencing the psoriasis-COPD link. Notably, in the fully weighted and fully adjusted model representing the general US population, the statistical significance of the association between psoriasis and COPD was no longer evident, indicating that their relationship may be complexly regulated by population characteristics and confounding factors.

Smoking is the primary risk factor for COPD development. Harmful components in tobacco smoke, such as nicotine, tar, and free radicals, can directly damage airway epithelial cells, triggering chronic airway inflammation, which in turn leads to airway remodeling, mucus hypersecretion, and progressive decline in lung function [24]. Multiple studies have shown that smoking is also a risk factor for psoriasis development [25–27]. For instance, a study involving 2,410 psoriasis patients reported that compared with never-smokers, former smokers had a 39% increased risk of developing psoriasis, and current smokers had a 94% increased risk. Moreover, the risk was positively correlated with the number of pack-years and duration of smoking [28]. Smoking cessation has been proven to reduce the risk of psoriasis onset; one study indicated that the risk of psoriasis was lower in quitters compared with persistent smokers [29]. The underlying mechanism may be related to smoking-induced systemic oxidative stress and inflammatory pathway activation. Smoking concurrently impairs the normal physiological functions of both the respiratory mucosal barrier and skin keratinocytes, which may be linked to the co-occurrence and progression of COPD and psoriasis. Based on this mechanism, smoking cessation serves as a cost-effective fundamental therapeutic measure for patients with both COPD and psoriasis. Currently, multiple national and international clinical guidelines list smoking cessation as a core intervention for COPD patients [30], and relevant studies have also confirmed that smoking cessation can improve skin lesion severity in psoriasis patients.

Obesity is another important confounding factor influencing the association between psoriasis and COPD. In recent years, multiple studies have confirmed that obesity can induce systemic chronic inflammation, a pathological mechanism that may be a key link connecting the co-occurrence risk of the two diseases [31–33]. Overweight patients with COPD have a higher mortality risk and are more prone to other comorbidities compared to those with normal weight [34]. Furthermore, a study on psoriasis patients showed that after a 10-week weight-loss dietary intervention, the average body weight decreased by 12%, accompanied by significant improvements in skin involvement area, pruritus severity, and quality of life [35].

Hypertension, as another significant confounding factor, may be linked to the comorbidity of both diseases through endothelial dysfunction induced by high blood pressure. In hypertension, the structure and function of vascular endothelial cells are impaired, leading not only to abnormal vasodilation and constriction but also to further activation of inflammatory signaling pathways, exacerbating the systemic chronic inflammatory state [36]. Chronic inflammation is both a core driver of airway remodeling and lung function deterioration in COPD [37, 38] and a key pathological basis for abnormal keratinocyte proliferation and persistent skin lesions in psoriasis [27, 39–41]. Furthermore, our study found a significantly enhanced association between psoriasis and COPD in individuals with poor marital status. This finding suggests that psychosocial factors may be involved in regulating the comorbidity of the two diseases. Chronic psychological stress arising from adverse marital relationships [42] can activate the sympathetic nervous system via the hypothalamic-pituitary-adrenal (HPA) axis, resulting in abnormal cortisol secretion, which in turn suppresses the normal function of immune cells and aggravates inflammatory responses [43]. This implies that when managing patients with both psoriasis and COPD, attention should be paid not only to controlling physiological risk factors such as smoking, obesity, and hypertension but also to assessing psychosocial status. Psychological interventions should be integrated when necessary. A multidimensional management strategy may improve the overall prognosis of these patients.

The regulatory patterns observed at the macro level require integration with micro-molecular characteristics to gain deeper insights into the biological basis of this comorbidity. In our GEO data analysis, we identified shared differentially expressed genes (DEGs), including UCK2, P4HA1, and HIBADH, as well as shared pathways such as the chemokine signaling pathway. These three DEGs showed significant expression abnormalities in both psoriasis and COPD patients. Moreover, diagnostic models constructed based on these genes effectively distinguished patients from healthy controls across different datasets, suggesting their potential as diagnostic biomarkers for the comorbidity. The chemokine signaling pathway, identified as a shared pathway, plays a crucial role. For example, CXCL8, by binding to its receptors CXCR1 and CXCR2, exerts potent activating and chemotactic effects on neutrophils. It serves as a core mediator of neutrophilic inflammation and a key regulator of neutrophilic airway inflammation in COPD [44]. Similarly, psoriatic lesions contain abundant chemokines that recruit and activate T lymphocytes, macrophages, and neutrophils, promoting the secretion of inflammatory cytokines and contributing to the initiation and maintenance of skin inflammation [45]. These specific molecular features may participate in systemic chronic inflammatory responses. Together with risk factors such as smoking, obesity, and hypertension, they may constitute a molecular-environmental network underlying the co-occurrence of the two diseases. However, the regulatory direction and causal relationships within this network require validation through prospective studies and causal inference methods. Psoriasis, with its readily observable skin phenotype, often prompts early patient concern and medical consultation. This characteristic provides a natural “window” for the early detection of COPD. Clinical practice could extend the routine examination items for psoriasis patients to include screening for genetic biomarkers. This approach would enable concurrent testing for COPD when patients present with skin issues, potentially identifying high-risk individuals at a subclinical stage before typical respiratory symptoms appear. This skin-disease-initiated screening model overcomes the limitations of traditional COPD screening, which relies heavily on pulmonary function tests. It may facilitate early detection and intervention for COPD, delaying disease progression, avoiding the high medical costs associated with mid-to-late-stage treatment, and significantly reducing the economic burden on patients and societal healthcare resource consumption.

Although psoriasis and COPD affect different organ systems, they share high homology in immune regulatory mechanisms. Systemic inflammation in psoriasis may be associated with pulmonary immune dysfunction or the initiation and exacerbation of lung inflammation. Numerous studies indicate that various pro-inflammatory cytokines, T-helper (Th) cell receptors and their co-stimulatory molecules, cytokines, chemokines and receptors, adhesion molecules, and proteases are involved in the pathophysiology of both COPD and psoriasis. Notably, dysfunctional Th1, Th17, and Th22 cells are key factors in psoriasis pathogenesis [46], while Th1 and Th17 cells are closely linked to COPD [47]. This common pathophysiological foundation provides a critical basis for exploring cross-disease treatment strategies. In recent years, phosphodiesterase 4 (PDE4) inhibitors have been used in the treatment of both COPD and psoriasis [48]. Building on this, for refractory COPD patients with inadequate responses to traditional bronchodilators and corticosteroids, therapeutic strategies borrowed from the well-established psoriasis field—such as biologic agents targeting shared inflammatory pathways (e.g., anti-IL-17 or anti-TNF-α monoclonal antibodies)—could be explored [49]. These biologics can precisely target inflammation pathways common to both diseases, potentially offering greater specificity and fewer side effects compared to conventional medications.

## Conclusion

The comorbidity association between psoriasis and COPD appears to be associated with risk factors such as smoking and hypertension, and is potentially regulated by shared differentially expressed genes and homologous immune-inflammatory pathways, indicating a multifactorial etiology. Utilizing psoriasis as a screening window, combined with genetic biomarkers, could enable the early identification and intervention of COPD. Furthermore, exploring cross-disease treatment strategies based on shared inflammatory pathways holds promise for improving patient prognosis and reducing the healthcare burden, offering new clinical perspectives.

### Limitations and future perspectives

Several limitations of this study should be acknowledged. First, due to its observational design, this study can only reveal associations between variables and cannot establish direct causality between aberrant gene expression and the disease comorbidity. The underlying regulatory mechanisms require validation through more rigorous causal inference approaches. Second, the lack of stratified analysis based on psoriasis subtypes (e.g., plaque, pustular) or COPD clinical phenotypes (e.g., emphysema-predominant, chronic bronchitis-predominant) may obscure potential heterogeneity in the observed associations. This could result in a lack of granularity in the conclusions drawn. Third, the diagnostic model based on the three-gene signature (UCK2, P4HA1, HIBADH) has not undergone external validation in independent, multi-center cohorts encompassing diverse ethnic populations. Consequently, its diagnostic performance and generalizability warrant further investigation.

Future studies should aim to address these limitations. Prospective cohort designs, coupled with Mendelian randomization analyses, could be employed to systematically evaluate the causal relationships linking gene expression, environmental risk factors, and the psoriasis-COPD comorbidity. Furthermore, integrating multi-omics technologies may offer deeper insights into gene-environment interactions and subtype-specific mechanisms, thereby strengthening the theoretical foundation for precise prevention and management strategies. Finally, conducting large-scale, multi-center clinical validation studies is essential to optimize the model’s performance and to facilitate its eventual translation into clinical practice.

## Data Availability

The datasets supporting the conclusions of this article are available in the NHANES and GEO repository, [https://wwwn.cdc.gov/nchs/nhanes/]; [https://www.ncbi.nlm.nih.gov/geo/].

## Abbreviations

AUC: Area Under the Curve
BMI: Body Mass Index
BP: Biological Processes
CCL: C-C Motif Chemokine Ligand
CCR: C-C Motif Chemokine Receptor
CC: Cellular Components
CI: Confidence Interval
COPD: Chronic Obstructive Pulmonary Disease
CXCR: C-X-C Motif Chemokine Receptor
DEGs: Differentially Expressed Genes
DTS: Decision Tree
FDR: False Discovery Rate
FEV₁/FVC: Forced Expiratory Volume in 1 s/Forced Vital Capacity
GEO: Gene Expression Omnibus
GBM: Gradient Boosting Machine
GPL: Gene Expression Omnibus Platform
HPA: Hypothalamic-Pituitary-Adrenal
IL: Interleukin
KEGG: Kyoto Encyclopedia of Genes and Genomes
KNN: K-Nearest Neighbor
LASSO: Least Absolute Shrinkage and Selection Operator
MF: Molecular Functions
NHANES: National Health and Nutrition Examination Survey
NCHS: National Center for Health Statistics
ns: Not Significant
OR: Odds Ratio
PDE4: Phosphodiesterase 4
PIR: Poverty Income Ratio
PSO: Psoriasis
RF: Random Forest
SD: Standard Deviation
SHAP: SHapley Additive exPlanations
TNF-a: Tumor Necrosis Factor-a
WGCNA: Weighted Gene Co-Expression Network Analysis
XGBoost: Extreme Gradient Boosting
